# Supplementary material for: Long-term continuous no-till corn-soybean systems: Examining soil carbon sequestration and nitrogen accumulation across various pools
Source: PLoS One. 2025 May 9;20(5):e0322891. doi: 10.1371/journal.pone.0322891 (PMC12064199; doi:10.1371/journal.pone.0322891)
Supplement: Attachment — Submitted filename: PONE-D-24-34295 reviewed.pdf [file pone.0322891.s002.pdf]

PLOS ONE

Long-term, Continuous No-Till Corn-Soybean Systems: Examining Soil Carbon Sequestration and Nitrogen Accumulation Across Various Pools

--Manuscript Draft--

**Manuscript Number:**	PONE-D-24-34295
**Article Type:**	Research Article
**Full Title:**	Long-term, Continuous No-Till Corn-Soybean Systems: Examining Soil Carbon Sequestration and Nitrogen Accumulation Across Various Pools
**Short Title:**	No-Till Impacts Soil Carbon and Nitrogen Dynamics
**Corresponding Author:**	Khandakar Rafiq Islam, Ph.D.<br>The Ohio State University<br>Piketon, OH UNITED STATES OF AMERICA
**Keywords:**	Microbial biomass; active carbon; particulate organic nitrogen; basal respiration; bulk density; fixed depth; equivalent mass
**Abstract:**	Tillage practices influence the soil's capacity as either a sink or source of carbon (C) within agroecosystems. An assessment spanning 6, 20, and 35 years of continuous no-till (NT) rainfed corn (Zea mays) - soybean (Glycine max) rotation with cereal rye (Secale cereale L.) as a cover crop practice showed significant increases in total microbial biomass (SMB by 1.4 to 2.1-fold), active C (by 11 to 33%), particulate organic C (POC by 1.4 to 2.7-fold), and total organic C (SOC by 14 to 69%) under NT compared to conventional tillage (CT). A similar impact of NT was observed on total N and particulate organic N (PON). While NT exhibited higher SMB pools and basal respiration (BR) rates, it significantly reduced (by 8 to 29%) specific maintenance respiration (qCO2) rates compared to CT. Ratios of passive C to active C increased under NT, indicating a higher proportion of non-labile C accumulation in SOC, which consequently resulted in decreased bulk density (ρb) compared to CT. This impact was more pronounced under long-term NT and on the surface than on subsurface soil. When using a fixed depth approach, SMB, active C, POC, and SOC were sequestered at 10.1 ± 4.1, 5.3 ± 1.8; 382.2 ± 55, and 587.4 ± 134.1 kg/ha/yr. in 0-15 cm depth and at 1.6 ± 0.5, 4.1 ± 1.4, 54 ± 8, and 192 ± 64 kg/ha/yr. in 15-30 cm depth. Likewise, PON and total N accumulation rates were 14.1 ± 5.5 and 72.2 ± 18.4 kg/ha/yr. at 0-15 cm depth and 4.3 ± 1.6 and 15 ± 5 kg/ha/yr. at 15-30 cm depth. A similar but variable rate of SOC sequestration and N accumulation was observed at both depths when using the equivalent mass compared to the fixed depth basis. Adjusting soil mass equivalence to account for ρb variability in fixed depth provides a more realistic estimation of SOC and N stocks, as fixed depth tends to overestimate these stocks. Our findings demonstrate that long-term NT practices consistently facilitate SOC sequestration and stoichiometric N accumulation. These benefits are distributed non-linearly across distinct SOC and N pools at surface depth and linearly at deeper depths within rainfed corn-soybean systems.
**Order of Authors:**	Maninder K. Khosa, PhD
	Kenan Barik, PhD
	Mohammad MR. Jahangir
	Nataliia O. Didenko
	Khandakar Rafiq Islam, Ph.D.
**Additional Information:**	
**Question**	**Response**
**Financial Disclosure**<br><br>Enter a financial disclosure statement that describes the sources of funding for the work included in this submission. Review	The author(s) received no specific funding for this work.

the submission guidelines for detailed requirements. View published research articles from *PLOS ONE* for specific examples.<br><br>This statement is required for submission and **will appear in the published article** if the submission is accepted. Please make sure it is accurate.<br><br>**Funded studies**<br>Enter a statement with the following details:<br>• Initials of the authors who received each award<br>• Grant numbers awarded to each author<br>• The full name of each funder<br>• URL of each funder website<br>• Did the sponsors or funders play any role in the study design, data collection and analysis, decision to publish, or preparation of the manuscript?<br><br>Did you receive funding for this work?	
**Competing Interests**<br><br>Use the instructions below to enter a competing interest statement for this submission. On behalf of all authors, disclose any competing interests that could be perceived to bias this work—acknowledging all financial support and any other relevant financial or non-financial competing interests.<br><br>This statement is required for submission and **will appear in the published article** if the submission is accepted. Please make sure it is accurate and that any funding sources listed in your Funding Information later in the submission form are also declared in your Financial Disclosure statement.<br><br>View published research articles from *PLOS ONE* for specific examples.	Not applicable

Powered by Editorial Manager® and ProduXion Manager® from Aries Systems Corporation

**NO authors have competing interests**<br><br>Enter: *The authors have declared that no competing interests exist.*<br><br>**Authors with competing interests**<br><br>Enter competing interest details beginning with this statement:<br><br>*I have read the journal's policy and the authors of this manuscript have the following competing interests: [insert competing interests here]*<br><br>* typeset	
**Ethics Statement**<br><br>Enter an ethics statement for this submission. This statement is required if the study involved:<br><br>• Human participants<br>• Human specimens or tissue<br>• Vertebrate animals or cephalopods<br>• Vertebrate embryos or tissues<br>• Field research<br><br>Write "N/A" if the submission does not require an ethics statement.<br><br>General guidance is provided below. Consult the submission guidelines for detailed instructions. **Make sure that all information entered here is included in the Methods section of the manuscript.**	Not applicable

**Format for specific study types**<br><br>**Human Subject Research (involving human participants and/or tissue)**<br>• Give the name of the institutional review board or ethics committee that approved the study<br>• Include the approval number and/or a statement indicating approval of this research<br>• Indicate the form of consent obtained (written/oral) or the reason that consent was not obtained (e.g. the data were analyzed anonymously)<br><br>**Animal Research (involving vertebrate animals, embryos or tissues)**<br>• Provide the name of the Institutional Animal Care and Use Committee (IACUC) or other relevant ethics board that reviewed the study protocol, and indicate whether they approved this research or granted a formal waiver of ethical approval<br>• Include an approval number if one was obtained<br>• If the study involved *non-human primates*, add *additional details* about animal welfare and steps taken to ameliorate suffering<br>• If anesthesia, euthanasia, or any kind of animal sacrifice is part of the study, include briefly which substances and/or methods were applied<br><br>**Field Research**<br><br>Include the following details if this study involves the collection of plant, animal, or other materials from a natural setting:<br>• Field permit number<br>• Name of the institution or relevant body that granted permission	
**Data Availability**<br><br>Authors are required to make all data underlying the findings described fully available, without restriction, and from the time of publication. PLOS allows rare exceptions to address legal and ethical concerns. See the PLOS Data Policy and FAQ for detailed information.	Yes - all data are fully available without restriction

Powered by Editorial Manager® and ProduXion Manager® from Aries Systems Corporation

A Data Availability Statement describing where the data can be found is required at submission. Your answers to this question constitute the Data Availability Statement and **will be published in the article**, if accepted.<br><br>**Important:** Stating 'data available on request from the author' is not sufficient. If your data are only available upon request, select 'No' for the first question and explain your exceptional situation in the text box.<br><br>Do the authors confirm that all data underlying the findings described in their manuscript are fully available without restriction?	
**Describe where the data may be found in full sentences. If you are copying our sample text, replace any instances of XXX with the appropriate details.**<br><br>• If the data are **held or will be held in a public repository**, include URLs, accession numbers or DOIs. If this information will only be available after acceptance, indicate this by ticking the box below. For example: *All XXX files are available from the XXX database (accession number(s) XXX, XXX.).*<br>• If the data are all contained **within the manuscript and/or Supporting Information files**, enter the following: *All relevant data are within the manuscript and its Supporting Information files.*<br>• If neither of these applies but you are able to provide **details of access elsewhere**, with or without limitations, please do so. For example:<br><br>*Data cannot be shared publicly because of [XXX]. Data are available from the XXX Institutional Data Access / Ethics Committee (contact via XXX) for researchers who meet the criteria for access to confidential data.*<br><br>*The data underlying the results presented in the study are available from (include the name of the third party*	Upon request, data will be available.

and contact information or URL).<br>• This text is appropriate if the data are owned by a third party and authors do not have permission to share the data.<br><br>* typeset	
Additional data availability information:	Tick here if the URLs/accession numbers/DOIs will be available only after acceptance of the manuscript for publication so that we can ensure their inclusion before publication.

Powered by Editorial Manager® and ProduXion Manager® from Aries Systems Corporation

Click here to access/download;Manuscript;COMPLETE MANUSCRIPT.doc

Long-term, Continuous No-Till Corn-Soybean Systems: Examining Soil Carbon Sequestration and Nitrogen Accumulation Across Various Pools

Maninder K. Khosa[1], Kenan Barik[2], Mohammad MR. Jahangir[3], Nataliia O. Didenko[4], Khandakar R. Islam[5]

[1]Dept. of Soils, Punjab Agricultural University, Ludhiana, India.

[2]Dept. of Soil Science and Plant Nutrition, Ataturk University, Erzurum, Turkey.
(Orcid no. 0000-0001-8147-0458).
[3]Dept. Soil Science, Bangladesh Agricultural University, Mymensingh, Bangladesh.
(orcid.org/0000-0002-9501-3239)
[4]Institute of Water Problems and Land Reclamation, Kyiv, Ukraine.
(orcid.org/0000-0002-0654-4231)
[5]Soil, Water and Bioenergy Resources, Ohio State University South Centers, Piketon, USA.
(Oorcid.org/0000-0002-2244-0395)

Corresponding author
Email: islam.27@osu.edu.

All the authors contributed equally and rationally to this work.

Abstract

Tillage practices influence the soil's capacity as either a sink or source of carbon (C) within agroecosystems. An assessment spanning 6, 20, and 35 years of continuous no-till (NT) rainfed corn (*Zea mays*) - soybean (*Glycine max*) rotation with cereal rye (*Secale cereale* L.) as a cover crop practice showed significant increases in total microbial biomass (SMB by 1.4 to 2.1-fold), active C (by 11 to 33%), particulate organic C (POC by 1.4 to 2.7-fold), and total organic C (SOC by 14 to 69%) under NT compared to conventional tillage (CT). A similar impact of NT was observed on total N and particulate organic N (PON). While NT exhibited higher SMB pools and basal respiration (BR) rates, it significantly reduced (by 8 to 29%) specific maintenance respiration (qCO_2) rates compared to CT. Ratios of passive C to active C increased under NT, indicating a higher proportion of non-labile C accumulation in SOC, which consequently resulted in decreased bulk density (ρb) compared to CT. This impact was more pronounced under long-term NT and on the surface than on subsurface soil. When using a fixed depth approach, SMB, active C, POC, and SOC were sequestered at 10.1 ± 4.1, 5.3 ± 1.8; 382.2 ± 55, and 587.4 ± 134.1 kg/ha/yr. in 0-15 cm depth and at 1.6 ± 0.5, 4.1 ± 1.4, 54 ± 8, and 192 ± 64 kg/ha/yr. in 15-30 cm depth. Likewise, PON and total N accumulation rates were 14.1 ± 5.5 and 72.2 ± 18.4 kg/ha/yr. at 0-15 cm depth and 4.3 ± 1.6 and 15 ± 5 kg/ha/yr. at 15-30 cm depth. A similar but variable rate of SOC sequestration and N accumulation was observed at both depths when using the equivalent mass compared to the fixed depth basis. Adjusting soil mass equivalence to account for ρb variability in fixed depth provides a more realistic estimation of SOC and N stocks, as fixed depth tends to overestimate these stocks. Our findings demonstrate that long-term NT practices consistently facilitate SOC sequestration and stoichiometric N accumulation. These benefits are distributed non-linearly across distinct SOC and N pools at surface depth and linearly at deeper depths within rainfed corn-soybean systems.

Key words: Microbial biomass, active carbon, particulate organic nitrogen, basal respiration, bulk density, fixed depth, equivalent mass.

Introduction

Conservation agriculture has the potential to rejuvenate and maintain soil quality by sequestering atmospheric CO_2 through plants into soil organic matter (SOM). Soil, which comprises 61% of the largest C pool in terrestrial ecosystems, faces continuous depletion due to human activities [1- 2]. Approximately 1.5 Pg CO_2 is emitted annually from deforestation, grassland degradation, and conventional agricultural practices, with annual tillage being a major contributor to SOC loss worldwide [3- 4].

Conventional tillage, a traditional method for land preparation and crop production, offers benefits such as soil inversion, mixing of crop residues, reduced surface compaction, increased air circulation, weed and disease control, and improved field workability [5- 6]. However, greater reliance on CT degrades soil quality by affecting biodiversity, disrupting structural stability, increasing decomposition of crop residue and native SOM, and accelerating erosion [6-7]. Increased CO_2 efflux and N release under CT result from the macroaggregate dispersion and subsequent mineralization of previously protected organic matter [8-9]. Additionally, increased O_2 diffusion and temperature rise due to soil and crop residue mixing following tillage further accelerate SOC mineralization by opportunistic microbes, thereby affecting the soil's biological, chemical, and physical properties [10-11].

Transitioning from CT to NT, one of the climate-smart agricultural practices, is effective to reduce operating costs, minimize erosion, conserve moisture, and sustain physical stability of soil [12-13]. Long-term NT practices are reported to enhance biodiversity and nutrient recycling, improve soil quality by accumulating SOC, and mitigate

climate change by reducing greenhouse gas emissions from agriculture [11-12, 15 change to 14]. In 2021, NT was practiced on over 44.5 million ha in the United States, up from 42 million ha in 2017 and 1.34 million ha in the early 1970s [15]. Globally, NT covered about 180 million ha, representing approximately 12.5% of total global cropland [13, 15]. The adoption of NT is increasing worldwide, driven by higher crop yields, farm economics, time savings, enhanced agroecosystem services, and potential C credits through SOC sequestration [15].

Soil organic carbon is considered one of the core indicators of soil quality, acting as a reservoir of substrates, energy, enzymes, and nutrients. It maintains biodiversity and efficiency, regulates biochemical reactions, supports physical stability and hydrological functions, and enhances agroecosystem resilience [5]. SOC comprises of diverse C pools, and considering it as a simple homogeneous pool ignores variations in its chemical composition, relative proportions, lability, biochemical turnover, and physico-chemical stability [16]. While detecting changes in SOC content takes several years, it is ~~more~~ meaningful to determine both labile and non-labile pools. These pools provide early and consistent indications of management-induced changes in SOC [16-17]. Labile SOC pools include microbial biomass C (SMB), active or permanganate oxidizable C, particulate organic C (POC), potentially mineralizable C (PMC), light-fraction C, cold and hot water extractable C, Anthrone reactive C, and fulvic acid C [2, 17]. Most SOC losses following CT originate from these pools [7, 17].

Several previous studies have reported that NT conversion from CT under humid or subhumid climates and with medium- and heavy-textured soils promotes SOC sequestration compared to CT [18-19]. In contrast, under warmer, dry climates and in sandy soils, NT is reported to sequester non-significant amounts of SOC compared to CT [19-20]. While most of these studies have shown that NT significantly increases SOC content in surface soil, but

CT maintains similar SOC levels near or below the 20-cm soil depth [11, 21-22]. However, other studies have reported that the downward movement of crop residues by earthworms and soil animals, leaching of dissolved organic carbon, and extended root growth may increase SOC concentration at deeper soil depths under long-term NT [18, 23]. Limited O_2 diffusion in moist, cooler, partially anaerobic soil under NT may restrict the decomposition of roots, facilitating their accumulation in SOC pools at subsurface depths. These studies report that NT significantly increased SOC along with SMB, active carbon, and POC contents at surface depth compared to CT.

Despite NT's importance in agroecosystem services, its impact on SOC sequestration is still debated when accounting for depth and bulk density (ρb) variations [3-4, 24]. Temporal variations in tillage operations that affect SOC content also impact the depth distribution of ρb. Because SOC content and ρb are impacted by changes in management practices, the commonly used fixed depth approach may become inaccurate due to changes in soil volume, leading to the overestimation or underestimation of SOC stocks [24-25]. Differences in SOC stocks associated with differences in ρb can be corrected using the equivalent soil mass approach, which varies only due to SOC concentration [26-28]. Recently, the use of equivalent soil mass for assessing SOC stock dynamics has been highlighted but is not widely implemented when considering SOC credit from croplands [24-25].

Despite comprehensive insights into SOC and stoichiometric N pools across agroecosystems, there remains a knowledge gap regarding the impact of continuous NT practices on specific pools or parameters of SOC and N such as SMB, biological properties, active and passive C, POC, and PON. Our hypothesis posits that under similar soil, crop rotation, and climatic conditions, long-term continuous NT will result in significant differences in SOC and N pools compared to CT when using the equivalent soil mass

approach. The objectives of our on-farm study were to evaluate the impact of continuous NT rainfed corn-soybean systems with cereal rye as a cover (0, 6, 20, and 35 yr.) on SMB and associated biological properties, PMC, active C, POC, SOC, PON, and total N contents. Additionally, we aimed to assess changes in ρb and quantify SOC sequestration and N accumulation in different pools compared to CT as a control.

Materials and Methods

Site description

The study was conducted at the 495-ha Brandt′s Family Farm in Carrol (39°50′915″ N, 82°41′579″ W), Fairfield County, in central Ohio, United States (**Fig. 1**). It is one of the oldest continuous NT and cover crops adapted farm in Ohio since 1971 [7]. The topography of the farm is moderate, with surface inclinations ranging between 1 and 3 degrees. Throughout the years, the farm site experiences a humid continental climate with four distinct seasons. Winters are cold and snowy, with temperatures averaging between -3.9°C and 0°C with snowfall averages 48.3 cm annually. Summers are hot and humid, with average temperatures ranging from 18.3°C to 26.7°C, with highest in July and lowest in January. Spring and fall offer milder temperatures. Rainfall is fairly consistent year-round, averaging around 102 cm, with spring being the wettest season.

Bennington silt loam (fine, illitic, mesic Aeric Epiaqualf) is the dominant soil across all sampled NT fields [29]. Averaged across NT duration, soil properties at a depth of 0 to 15 cm include a pH of 5.6 to 6.2, electrical conductivity (ECe) of 158 to 283 dS/m, cation exchange capacity (CEC) of 15 to 24 meq/100 g, available nitrogen (AN) of 17.8 to 27.1 mg/kg, available phosphorus (AP) of 10 to 24 mg/kg, sand 23 to 26%, silt 58 to 61%, and

clay 15 to 17%. At a depth of 15 to 30 cm, soil properties include a pH of 5.5 to 5.9, ECe of 17 to 21 dS/m, CEC of 16 to 21 meq/100 g, AN 6 to 14 mg/kg; AP 5 to 9 mg/kg; sand 20 to 22%, silt 59 to 60%, and clay 18 to 21%.

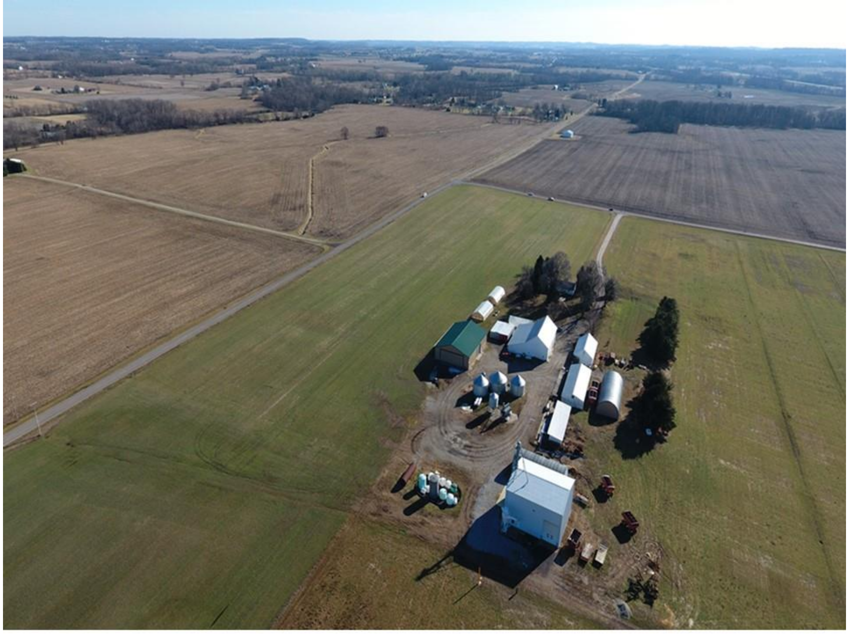

Fig. 1. Aerial view of the Brandt's family farm at Carroll, Fairfield county, Oho, USA.

Soil sampling and processing

Composite soil samples were collected from fields with six (NT_6), twenty (NT_{20}), and thirty-five (NT_{35}) years of continuous NT and their adjacent CT fields (NT_0). The crop rotation was a rainfed corn-soybean system with cereal rye as a winter cover crop used at all sites. The NT fields utilized a planter with a wavy coulter to open a 5 cm wide path for planting crops without disturbing the surface residues. In contrast, the CT plots were chisel plowed annually in the fall. All plots received standard chemical fertilization and weed control practices. Nitrogen fertilization was performed using 28% urea-ammonium nitrate (UAN) at 160 L/ha at planting, followed by 28% UAN at 455 L/ha as a side-dress. Phosphorus and potassium fertilizers were broadcast annually at 100 kg/ha using 0-46-0 and 0-0-61 chemical fertilizers in the previous fall.

Four sampling plots (20 m x 20 m) were GPS-referenced as pseudo-replicates in each tillage treatment before soil sampling. Six cores were collected from a depth of 0-30 cm (segmented into 0-15 and 15-30 cm) using a Giddings soil probe from each of the geo-referenced pseudo-replicated plots, following a systematic sampling procedure (4 NT treatments x 4 pseudo-replicated plots x 2 depths x 6 cores = 192 cores). The cores were pooled and mixed to obtain a composite sample at each depth and placed in sealable plastic bags (4 NT treatments x 4 pseudo-replicated plots x 2 depths = 32 samples). While a sub-sample of the soil was sieved to 2 mm prior to analysis for SMB and associated biological properties, another portion of the soil was air-dried in the shade at room temperature (~25°C) for 15 days before selected chemical and physical analysis.

Soil analysis

The SMB was determined using a rapid microwave irradiation and extraction method [30]. Basal respiration (BR), as a measure of antecedent biological activity, was determined using *in vitro* static incubation of unamended field-moist soil at 25±1°C in a temperature-controlled incubator for a period of 15 days [8]. The specific maintenance respiration (qCO_2) rates was calculated as BR per unit of SMB per day [31]. PMC was calculated by dividing the total amount of CO_2 evolved from the soil during the 15 days *in vitro* incubation by the SOC content [5]. The SOC and total N contents were measured on finely ground (<200 µm) air-dried soils using the dry combustion method with a FlashEA-1112 series CNHS-O analyzer. Active C, or permanganate oxidizable C, was determined spectrophotometrically based on mild oxidation of SOC in 2-mm sieved air-dried soil with a pH neutral 0.02M $KMnO_4$ solution [17]. Passive C was calculated by subtracting active C from SOC. The SMB: SOC ratio, also known as the microbial quotient (qR), was calculated [31].

To determine POC and PON contents, free and aggregate-protected particulate organic matter (POM) was collected after dispersing a 10 g sample of 2-mm air-dried soil

with small glass marble in 50-mL screw-top polypropylene tubes by shaking with 25 mL of 0.5% Na-hexametaphosphate solution at 250 rpm for 18 hr. [32]. After shaking, the dispersed soil suspensions were passed through a 53-μm sieve, washed slowly under running water to collect sand-associated POM, and oven-dried at 65±2°C using a forced-air oven until a constant weight was obtained. A sample of sand-associated POM was burned at 480±10°C for 4 hr. in a muffle furnace to calculate POM content by loss on ignition. Another sample of finely ground sand-associated POM was placed in Zn capsules and analyzed for POC and PON concentrations using the FlashEA-1112 series CNHS-O analyzer. Results were expressed on a sand-free basis.

Soil pH was determined in 1:2.5 soil-distilled deionized water slurries using a combination glass electrode. The ECe was determined in 1:1 soil-distilled deionized water using a standard conductivity meter. Antecedent soil moisture content was determined thermogravimetrically after oven-drying the field-moist soil at 105±2°C for 24 hr. until a constant weight was obtained. The AN with 2M KCl extraction and Mehlich-3 AP were determined by Shimadzu® TOC/TOC CN analyzer and Integrated Coupled Plasma Spectrophotometry, respectively. Soil CEC was determined following the ammonium acetate extraction and Shimadzu® total dissolved CN analyzer. Soil ρb was calculated by the standard core method from the relationship of oven-dried mass of a known volume of soil. Particle size analysis for sand, silt, and clay contents was determined by the Bouycous hydrometer method.

Soil carbon sequestration and nitrogen accumulation

Using the fixed depth approach, the stocks of SMB, active C, POC, passive C, SOC, PON, and total N at different soil depths were calculated by multiplying their respective concentrations with the sampling depth (d) and concurrently measured ρb values as follows:

$$\text{Fixed depth stock (Mg/ha)} = (\rho b \times d \times C \text{ or } N \text{ pools} \times 10000)$$

While soils are susceptible to changes in ρb in response to transitional or long-term tillage practices, an increase or decrease in ρb invariably results in a corresponding increase or decrease in soil mass for the same depth over time. Therefore, it is necessary to adjust for soil depth correction to address random errors associated with ρb variations. Accordingly, a depth correction factor (d_{eq}) was calculated by dividing the lowest ρb value by the ρb values of all treatments prior to converting the SOC and N stocks in equivalent mass basis [28]:

$$\text{Equivalent mass stock (Mg/ha)} = (\rho b \times C \text{ or } N \text{ pools} \times 10000) \times d_{eq} \text{ factor}$$

To assess NT soil's capacity to sequester SOC and accumulate N in different pools at 0-15 and 15-30 cm depths, fixed depth or equivalent mass stocks of SMB, active C, POC, passive C, SOC, PON, and total N was plotted as a function of continuous NT duration (0, 6, 20, and 35 yr.) using best-fit linear and non-linear regression models.

The equivalent mass of SMB, active C, POC, passive C, SOC, PON, and total N stocks were regressed against their fixed depth counterparts to account for their variability and potential overestimation or underestimation in predicting the NT soil's temporal capacity to sequester SOC and accumulate N in different pools.

Statistical analysis

Significant differences in SOC and N pools and other soil properties attributed to the effects of years of NT were assessed in a factorial arrangement (4 temporal NT treatments × 2 soil depths) using the two-way analysis of variance procedure in SAS®. Tillage and depth were considered fixed predictor variables. Main and interactive effects of tillage and depth on dependent variables with a value of $p \leq 0.05$ were considered significant unless otherwise noted. Best-fitted linear and non-linear regression models were used to calculate SOC

sequestration and N accumulation rates in different pools at various depths, determined using SigmaPlot®.

Results and Discussion

Changes in soil organic carbon and nitrogen pools

The SMB, a living component of SOC, was significantly impacted by both NT duration and depth, without significant interaction between them (**Table 1**). Averaged across soil depths, the highest SMB was observed under NT_{35}, followed by NT_{20} and NT_6, compared to CT (N_{T0}), with increases of 1.4, 1.5, and 2.1-fold, respectively. The qR (SMB: SOC), representing the biologically active pool of SOC, increased variably, with the highest values under NT_6, followed by NT_{20} and NT_{35}, and the lowest under CT. BR rates increased by 29%, 31%, and 43% under NT_6, NT_{20}, and NT_{35}, respectively, compared to CT. In contrast, qCO_2, a measure of microbial catabolism (C used per unit of SMB per day), was higher under CT and reduced by 7.9%, 15.6%, and 29.4% under NT_6, NT_{20}, and NT_{35}, respectively. PMC showed the highest values under NT_6 and the lowest under long-term NT compared to CT.

Table 1. Long-term continuous no-till effects (0, 6, 20, and 35 yr.) on total microbial biomass (SMB), metabolic quotient (qR), basal (BR) respiration and specific maintenance (qCO_2) respiration rates, and potentially mineralizable carbon (PMC) at different soil depths under a rainfed corn-soybean system with cereal rye as a winter cover crop.

Tillage	Depth	SMB	qR	BR	qCO_2	PMC
Trt.	(cm)	(mg/kg)	(%)	(mg/kg/d)	(µg/mg/d)	(%)
NT_0 (CT)	0-30	123.2c$^{\neq}$	1.06c	14.7b	117.8a	1.83b

NT_6	0-30	176.1b	1.32a	19a	108.5b	2.16a
NT_{20}	0-30	189.3b	1.16b	19.3a	99.4c	1.7bc
NT_{35}	0-30	248.2a	1.2b	21a	83.2d	1.5c
Tillage x soil depth						
NT_0 (CT)	0-15	139.3^{ns}	0.9^{ns}	18.7^{ns}	135.9^{ns}	1.81^{ns}
	15-30	107.1	1.23	10.7	99.7	1.85
NT_6	0-15	222.9	1.2	23.3	106.1	1.89
	15-30	129.3	1.42	14.7	110.8	2.43
NT_{20}	0-15	246.2	1.13	27.3	111.1	1.88
	15-30	132.4	1.18	11.3	87.6	1.51
NT_{35}	0-15	334.4	1.22	29.3	87.6	1.61
	15-30	162	1.18	12.7	78.8	1.39

≠ Treatment means under each column separated by same lower-case letters (a, b, c, and d) were not significantly different among years of NT duration at $p \leq 0.05$.

NT treatments had significantly higher SOC content (by 14 to 69%) than CT, with the highest under NT_{35} (**Table 2**). Total N content showed a similar trend. NT also increased both active- and passive C contents over CT, with more passive C accumulating under long-term NT. Both POC and PON progressively increased under NT compared to CT. The lowest increase in POC was 1.4-fold under NT_6 and the highest was 2.7-fold under NT_{35}. SOC as POC increased by 27.8% to 35.4% under NT compared to 22% under CT. Similarly, PON increased, with total N as PON ranging from 12.2% to 16% under NT compared to 8.2% under CT.

Table 2. Long-term continuous no-till effects (0, 6, 20, and 35 yr.) on total organic carbon (SOC), active carbon (AC), particulate organic carbon (POC), particulate

organic nitrogen (PON) and total nitrogen (TN) contents at different soil depths under a rainfed corn-soybean system with cereal rye as a winter cover crop.

Tillage Trt.	Depth (cm)	SOC (g/kg)	AC (mg/kg)	POC	TN	PON	PC: AC	POC: PON
				(g/kg)				
NT$_0$ (CT)	0-30	12.1c[≠]	454.3c	2.66d	1.12c	0.122d	25.6b	22.1a
NT$_6$	0-30	13.8c	504.2b	3.84c	1.3bc	0.158c	26.4b	24a
NT$_{20}$	0-30	16.5b	588.1a	5.57b	1.53b	0.238b	27.1b	23.5a
NT$_{35}$	0-30	20.5a	603.9a	7.27a	1.79a	0.287a	30.8a	22.5a
Tillage x soil depth								
NT$_0$ (CT)	0-15	15.5^{ns}	504.9^{ns}	3.9^{ns}	1.34^{ns}	0.183^{ns}	29.7^{ns}	21.3^{ns}
	15-30	8.7	403.7	1.42	0.9	0.062	20.6	22.9
NT$_6$	0-15	18.6	601.6	5.93	1.76	0.241	29.9	24.6
	15-30	9	406.9	1.75	0.83	0.075	21.1	23.4
NT$_{20}$	0-15	21.8	648.1	8.69	2	0.373	32.6	23.3
	15-30	11.2	528.2	2.44	1.07	0.103	20.2	23.6
NT$_{35}$	0-15	27.3	671.8	10.2	2.31	0.452	35.7	22.3
	15-30	13.7	535.9	2.76	1.27	0.122	24.6	22.6

[≠] Treatment means under each column separated by same lower-case letters (a, b, c, and d) were not significantly different among years of NT duration at $p<0.05$

Significantly higher SMB content and efficient biological activities, along with increased SOC and N in different pools under long-term NT compared to CT, were associated with a diversified microbial food web [33-35]. The microbial diversity, especially a fungi-dominated food web, resulted from reduced contact between microbes and the surface placement of unfragmented crop residues, greater root growth, and chemical

fertilization [33, 35]. Fungal food webs are energy-efficient but slow decomposers, favoring longer retention of crop residues and increasing SOC content under undisturbed, cooler, moist and partially anerobic NT conditions [33, 36].

The higher energy efficiency of fungal food webs under long-term NT supported a larger SMB pool, but lower qCO_2 rates compared to CT [8, 22, 34]. Lower qCO_2 values indicate more labile C is assimilated into SMB cells rather than respired. This efficient anabolism explains higher SMB, active C, BR, and lower qCO_2 values under long-term NT.

Increased SOC content in different pools under NT stoichiometrically increased total N content, most visible in higher POC and PON values [37]. Both POC and PON pools, derived from crop residue, especially roots and microbially-processed C and N metabolites, were involved in soil macroaggregate formation and get themselves protected from microbes [11, 18, 36]. NT resulted in greater surface stratification of labile C and nutrients, providing food and energy sources that enhanced SMB growth and activity compared to deeper soils. A significantly higher content of both POC and PON at NT surface is due to increased root biomass distribution [34, 38]. In contrast, higher qCO_2 values at deeper soil depths result from increased microbial catabolism due to limited availability of labile C and N substrates.

Long-term residue retention at the surface under NT leads to exposure to sunlight and UV radiation, which alters residue quality and slows decomposition, resulting in a higher proportion of passive C to active C accumulation in SOC [18, 39]. This results in a higher passive C to active C ratio in SOC under long-term NT, supporting our findings. The passive C is a biochemically processed and physically protected pool of SOC, with less susceptibility and accessibility to SMB decomposition [36, 40-41]. It is suggested that the greater stabilization of SOC is associated with microbially-processed belowground C metabolites derived from roots [41].

Changes in soil bulk density

Bulk density (ρb) was significantly influenced by years of NT (**Fig. 2a**). Initially, ρb increased by 3.4% under transitional NT (NT_6), then decreased by 4.7% under NT_{20} and 7.1% under NT_{35}. This decrease was more pronounced in surface soil than in sub-surface soil. The relationship of ρb with NT duration accounted for 53% and 88% of the variability in ρb changes at 0-15 and 15-30 cm depths, respectively. In addition, when ρb values were regressed on SOC contents, the non-linear relationship accounted 82% of the variations in decreasing ρb by increasing SOC content under long-term NT (**Fig. 2b**).

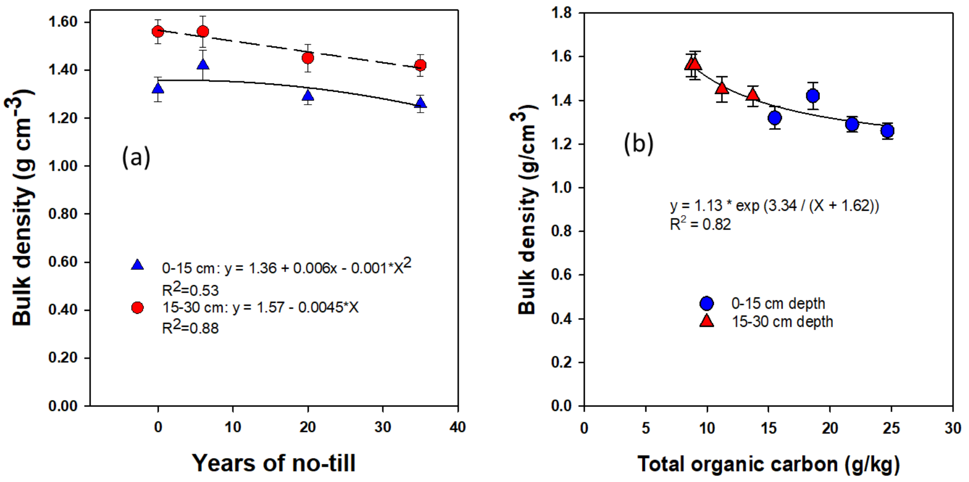

Fig. 2. Long-term continuous no-till effects (0, 6, 20, and 35 yr.) on (a) bulk density and (b) the relationship between bulk density and total organic carbon at different soil depths under a rainfed corn-soybean system with cereal rye as a winter cover crop. Treatment mean values are presented along with the standard error of the mean.

Increased ρb under transitional NT is due to temporary consolidation and compaction of soil by natural processes [18]. However, long-term NT reverses this effect by increased SOC content followed by macropore formation by soil fauna especially earthworms and plant roots [42]. Previous studies report a 10% increase in ρb under transitional NT, but long-term NT shows comparable or lower ρb than CT due to increased SOC content [18]. SOM, as the

prime source of SOC, with its low ρb values (0.5 to 0.6 g/cm³) and ability to enhance aggregate formation, increases macroaggregation and decreases ρb under long-term NT [43]. An increase in subsurface soil ρb compared to surface soil was anticipated due to lower SOC contents. This expectation is supported by a significant non-linear decrease in ρb corresponding with an increase in SOC content under long-term NT management, corroborating findings from previous NT studies.

Changes in soil carbon and nitrogen sequestration and accumulation

The stocks of SOC and N in different pools, calculated by multiplying their concentrations with depth and ρb using fixed depth and equivalent mass approaches, were significantly influenced by NT duration (**Fig. 3-6**). Based on the fixed depth approach, the SOC and N stocks showed surface stratification; however, the differences in their stocks gradually diminished under long-term NT, with a significant exponential rise toward a plateau over the years. A similar or slightly variable impact of NT on SOC and N stocks was observed at both depths when using the equivalent mass compared to the fixed depth basis.

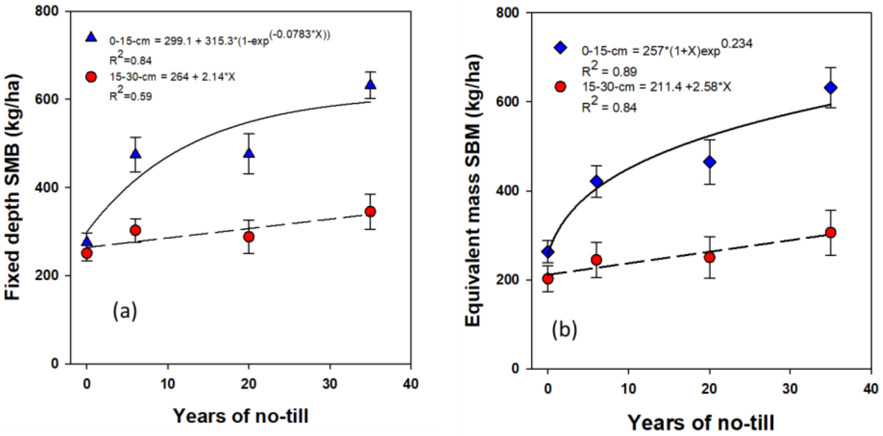

Fig. 3. Long-term continuous no-till effects (0, 6, 20, and 35 yr.) on total microbial biomass carbon (SMB) stocks at various depths under a corn-soybean system with

cereal rye as a winter cover crop: Fixed-depth and equivalent mass approaches. Treatment mean values are presented with the standard error of the mean.

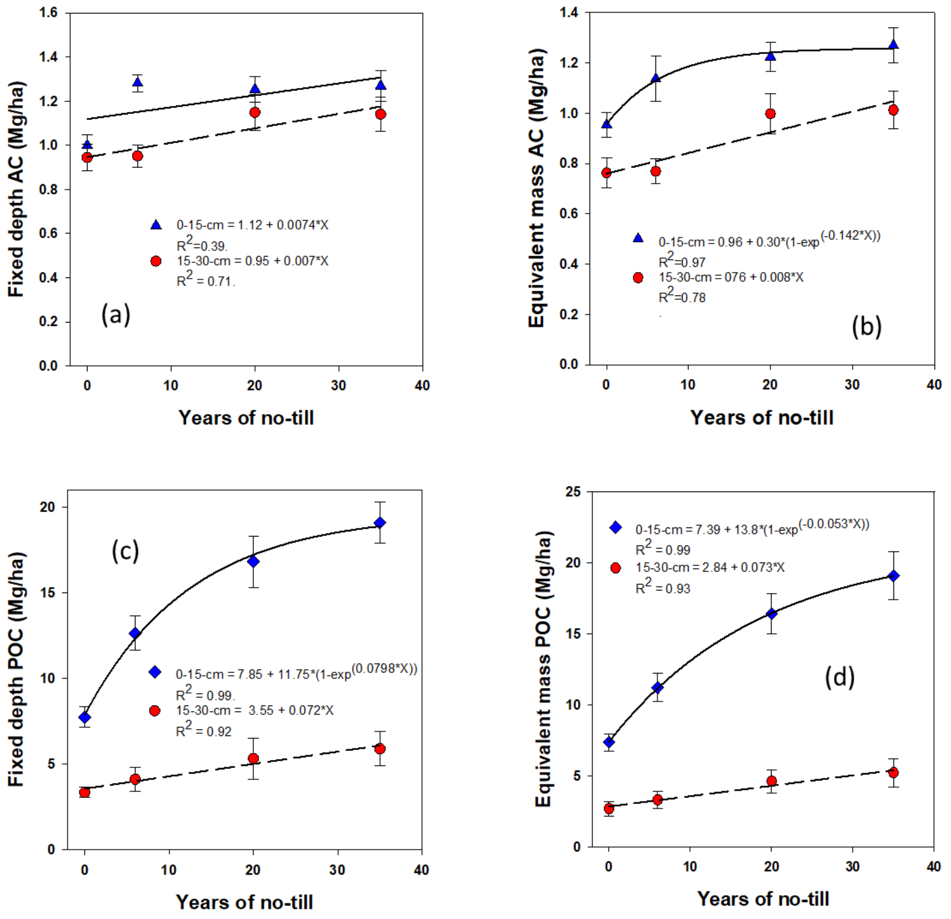

Fig. 4. Long-term continuous no-till effects (0, 6, 20, and 35 yr.) on active carbon (AC) and particulate organic carbon (POC) sequestration at different depths under a rainfed corn-soybean system with cereal rye as winter cover crop: Fixed-depth and equivalent mass approaches. Treatment mean values are presented with the standard error of the mean.

Based on a fixed depth basis, SMB was sequestered non-linearly at 10.1±4.1 kg/ha/yr. at 0-15 cm and linearly at 1.6±0.5 kg/ha/yr. at 15-30 cm (**Fig. 3a**). When using equivalent mass, SMB sequestration was 11.9±5.1 kg/ha/yr (non-linear) at 0-15 cm and 1.9±0.6 kg/ha/yr. (linear) at 15-30 cm (**Fig. 3b**). In contrast, active C sequestered linearly by 5.3±1.8 and

4.1±1.4 kg/ha/yr. at 0-15 and 15-30 cm, respectively, based on fixed depth approach (**Fig. 4a**). Using equivalent mass, active C was sequestered non-linearly at 12.8±6 kg/ha/yr. in surface soil and linearly at 6±2 kg/ha/yr. in subsurface soil (**Fig. 4b**). The rates of POC sequestration exhibited non-linear responses (382.2±155.4 vs. 352.5±132.1 kg/ha/yr) at a depth of 0-15 cm and linear responses (54±18 vs. 54.8±18.3 kg/ha/yr) at a depth of 15-30 cm for both fixed depth and equivalent mass (**Fig. 4c & 4d**).

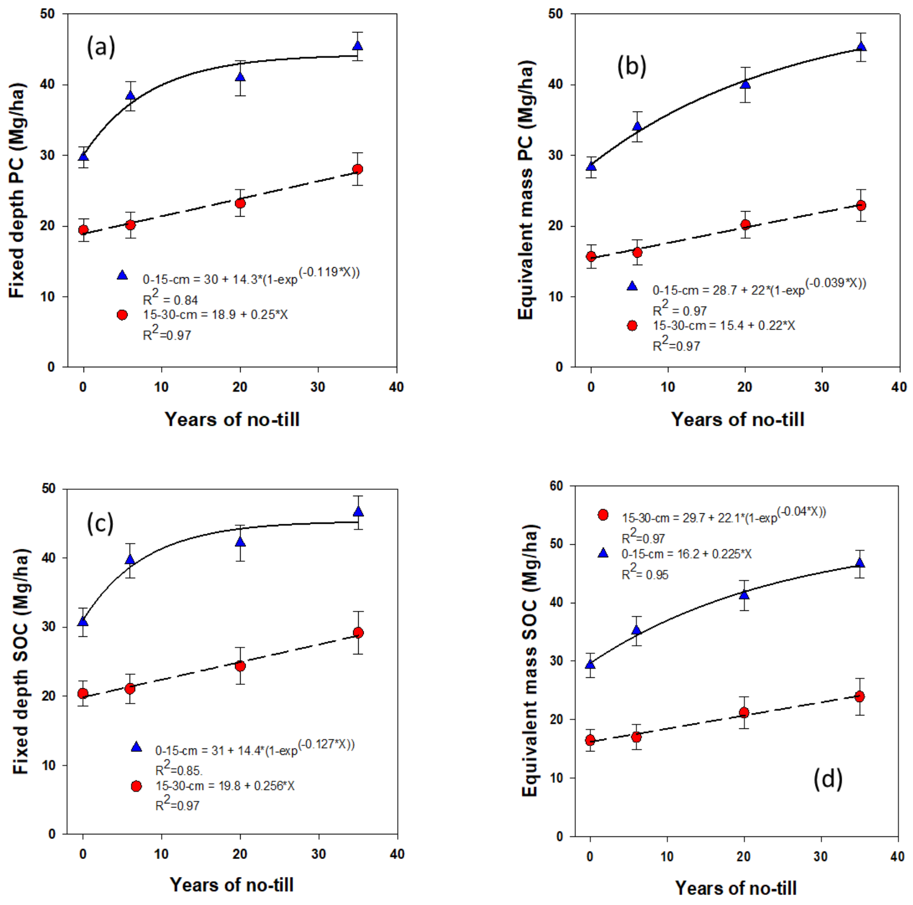

Fig. 5. Long-term continuous no-till effects (0, 6, 20, and 35 years) on passive carbon (PC) and total organic carbon (SOC) stocks at different depths under a rainfed corn-soybean system with cereal rye as winter cover crop: Fixed-depth and equivalent mass approaches. Treatment mean values are presented with the standard error of the mean.

Based on fixed depth, passive C and SOC were sequestered non-linearly (566.8±127.2 and 587.4±134.1 kg/ha/yr.) at 0-15 cm and linearly at 187.5±62.5 and 192±64 kg/ha/yr. at 15-30 cm (**Fig. 5a & 5c**). When using equivalent mass, both passive C and SOC sequestration rates were observed to be 457.2±164.1 and 467.5±168.3 kg/ha/yr at a depth of 0-15 cm, and 161.3±53.8 and 168.8±56.3 kg/ha/yr at a depth of 15-30 cm (**Fig. 5b & 5d**). PON and total N accumulated non-linearly at rates of 14.1±5.5 and 72.2±18.4 kg/ha/yr, respectively, at a depth of 0-15 cm, and linearly at rates of 4.3±1.6 and 15±5 kg/ha/yr, respectively, at a depth of 15-30 cm, based on the fixed depth approach (**Fig. 6a & 6c**). Using the equivalent mass approach, PON and total N accumulated similarly at rates of 15.6±5.7 and 52.4±19.9 kg/ha/yr, respectively, at 0-15 cm and 2.5±0.8 and 17.3±5.8 kg/ha/yr, respectively, at 15-30 cm (**Fig. 6b & 6d**).

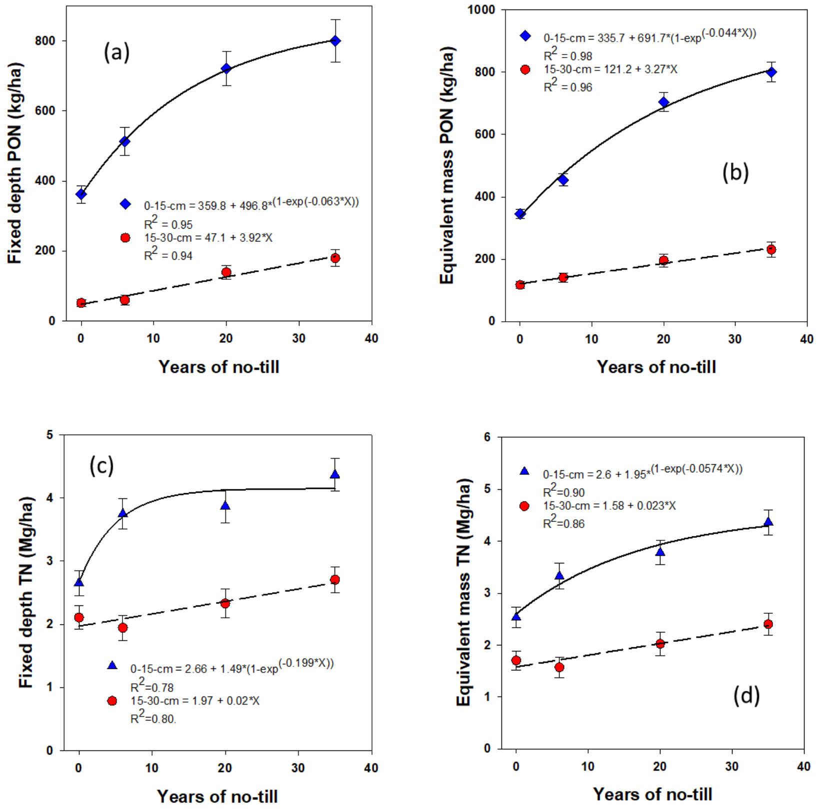

Fig. 6. Long-term continuous no-till effects (0, 6, 20, and 35 years) on particulate organic nitrogen (PON) and total nitrogen (TN) stocks at different depths under a

rainfed corn-soybean system with cereal rye as winter cover crop: Fixed-depth and equivalent mass approaches. Treatment mean values are presented with the standard error of the mean.

SOC and N stocks in different pools showed significant linear relationships using both fixed depth and equivalent mass approaches, with fixed depth consistently overestimating values (**Fig. 7 - 9**). The SMB stock relationship was highly significant (R^2=0.98) with a slope of 1.09±0.056 ($p<0.0001$); however, fixed depth overestimated SMB by 65.8±22.5 kg/ha ($p=0.027$) compared to equivalent mass (**Fig. 7**). Similar overestimations were observed for active C, POC, passive C, SOC, PON, and total N stocks (**Fig. 8 & 9**).

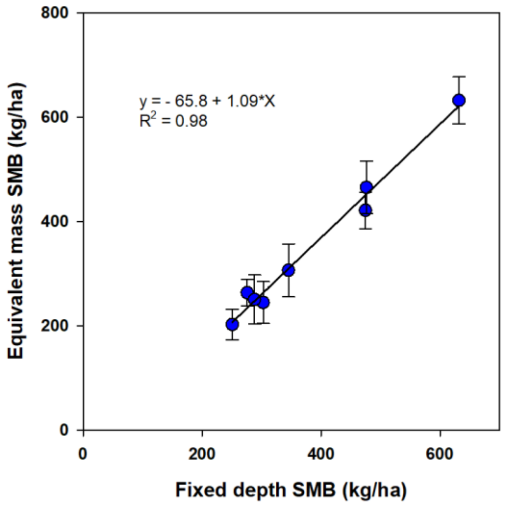

Fig. 7. Relationship between total microbial biomass carbon (SMB) stocks under a rainfed corn-soybean system with cereal rye as winter cover crop: Fixed-depth and equivalent mass approaches. Treatment mean values are presented with the standard error of the mean.

Fixed depth active C stock accounted for 88% of the variations in equivalent mass active C stock with a slope of 1.26±0.18 ($p=0.004$), overestimating by 40.4±10.1 kg/ha at

p=0.09 (**Fig. 8a**). As expected POC was overestimated by 842±142 kg/ha (p=0.025) with R^2=0.99 and slope 1.02±0.03 at p<0.0001 (**Fig. 8b**). Likewise, passive C stock was significantly overestimated by 6.2±1.9 Mg/ha (p=0.017) with R^2=0.98 and a slope of 1.11±0.06 at p<0.0001 (**Fig. 8c**). The SOC stock relationship was highly significant (R^2=0.98) with a slope of 1.12±0.06 (p<0.0001); however, fixed depth overestimated by 6.61±2.01 Mg/ha at p=0.017 (**Fig. 8d**). Fixed depth PON stock accounted for 99% of the variations in equivalent mass PON stock (slope 1.03±0.024, p<0.0001), overestimating by 37.8±11.3 kg/ha at p=0.015 (**Fig. 9a**). Total N stock showed a similar relationship (R^2=0.98, slope 1.11±0.06, p<0.0001), with fixed depth overestimating by 576.5±175 kg/ha at p=0.016 (**Fig. 9b**).

It is reported that SOC storage capacity is finite and significantly influenced by the content of silt and clay, as well as their complex interactions with reactive minerals and metal (oxyhydr)oxides [44-45]. These factors impact the soil's ability to retain SOC over time. Consequently, SOC sequestration and N accumulation in different pools plateau towards the soil's capacity is reached, especially at the surface under long-term NT. Our results showed that at 0-15 cm depth, SOC sequestration and N accumulation plateaued after about 15 yr. of NT, whereas at 15-30 cm depth, they continued to increase linearly, suggesting further accumulation even beyond 35 yr. While other studies report minor or insignificant NT impact on SOC sequestration [3, 20, 45-46], our results align with those indicating SOC sequestration and stoichiometric N accumulation under diverse conditions [14, 21, 27, 47].

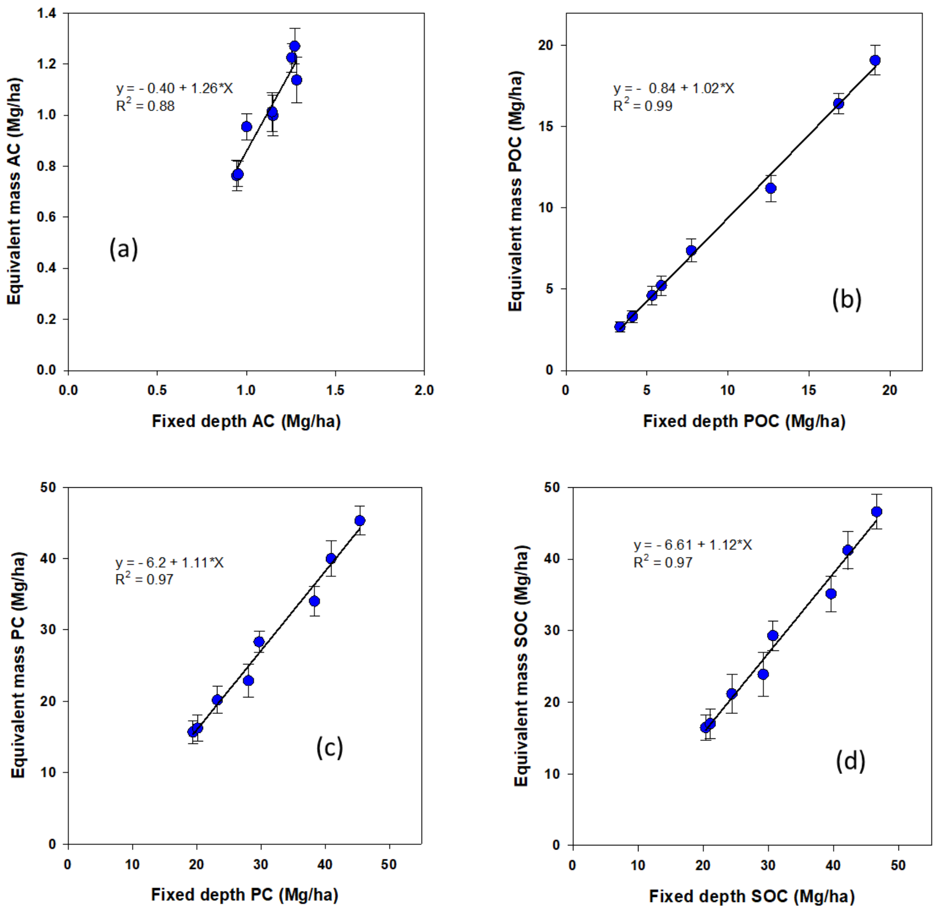

Fig. 8. Relationship between active carbon (AC), particulate organic carbon (POC), passive carbon (PC), and total organic carbon (SOC) stocks under a rainfed corn-soybean system with cereal rye as winter cover crop: Fixed-depth and equivalent mass approaches. Treatment mean values are presented with the standard error of the mean.

Several studies observed that switching from plowing to NT increased SOC stock by 570 kg/ha/yr.; while other studies reported that SOC sequestration linearly peak between > 0 and 10 yr. and decline after 15 to 20 yr. [18, 21, 48]. It is reported that sequestration rates of biological pool of SOC (SMB) were 22, 13, 7, and 3 kg/ha/yr. at the 0-7.5, 7.5-15, 15-22.5, and 22.5-30-cm depths, respectively for the first 10 yr. of NT [18]. During the same time, the macroaggregate protected C sequestration rates were 92, 63, 47, and 37 kg/ha/yr., respectively, at the same depths. By 20 yr. of NT, SMB and macroaggregate protected C

sequestration rates plateaued at the surface. Similarly, another study reported that SOC sequestration under NT decreased over time with initially higher rates at 740 kg/ha/yr. in the 3-10 yr. followed by 260 kg/ha/yr. in the 11-20 yr., and 95 kg/ha/yr. for longer than 20 yr. in the medium textured prairie soils in Canada [49].

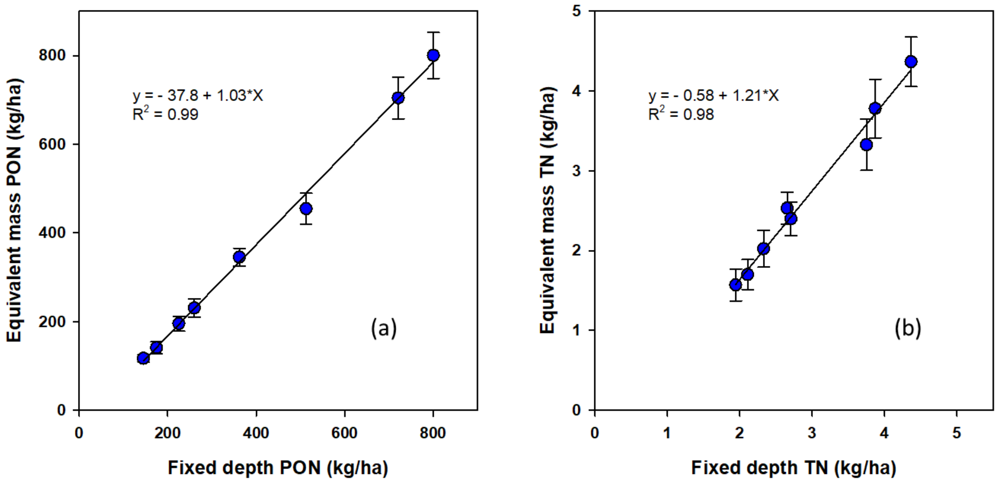

Fig. 9. Relationship between particulate organic nitrogen (PON) and total nitrogen (TN) stocks under a rainfed corn-soybean system with cereal rye as winter cover crop: Fixed-depth and equivalent mass approaches. Treatment mean values are presented with the standard error of the mean.

Several studies reported that equivalent mass SOC and N stocks are more realistic, with fixed depth often lead to inaccuracies [26-28, 50]. However, it has been suggested that the fixed-depth approach overestimates SOC sequestration when associated with soil compaction (higher ρb) and underestimates due to lower ρb associated with soil expansion [51]. Our results validates that adjusting soil mass equivalence provides more realistic SOC and N stocks in different pools, as fixed depth overestimates them.

Conclusions

Long-term continuous NT practice in a rainfed corn-soybean rotation with cereal rye as a winter cover crop significantly increased SMB, active carbon, POC, passive C, SOC, PON, and total N. It also improved biological efficiency (reduced qCO_2) compared to CT. These positive effects were more evident in the surface soil than in the subsurface soil. A higher proportion of passive C compared to active C in SOC suggests an increase in the stable form of SOC under NT. While transitional NT resulted in slightly higher compaction (ρb), the soil became more porous under long-term NT at both depths compared to CT. SOC sequestration and stoichiometric N accumulation in different pools under NT were nonlinear at the surface and showed a linear response at subsurface depth when calculated using both fixed depth and equivalent mass approaches. Our on-farm NT study has demonstrated that adjusting for soil mass equivalence provides a more realistic estimation and prediction of SOC sequestration and N accumulation in different pools under long-term NT.

Declaration of Competing Interest

The authors declare that they have no known competing financial interests or personal relationships that could have appeared to influence the work reported in this paper. They affirm that their research has been conducted with the utmost integrity and objectivity, free from any external pressures or biases that could potentially affect the results or conclusions.

Acknowledgement

The authors extend their deepest gratitude to the late David Brandt, the esteemed owner of Brandt Family Farm located in Carroll, Fairfield County, in central Ohio, United States. His

generosity in allowing access to his farm and providing comprehensive logistical support for soil sampling was invaluable to this research. David Brandt's contributions and commitment to sustainable agriculture have left a lasting legacy, and his support has been crucial in advancing our understanding of no-till and soil health.

References

1. Scharlemann JPW, Tanner EVJ, Hiederer R, Kapos, V. Global soil carbon: Understanding and managing the largest terrestrial carbon pool. Carbon Management. 2014; 5: 81-91.
2. Islam KR, Dick WA, Watts DB, Gonzalez JM, Fausey NR, Flanagan NC, Reeder RC, VanToai TT, Batte MT. Gypsum, crop rotation, and cover crop impacts on soil organic carbon and biological dynamics in rainfed transitional no-till corn-soybean systems, PLOS ONE. 2022; 17: 9 (e0275198).
3. Baker JM, Ochsner TE, Venterea RT, Griffis TJ. Tillage and soil carbon sequestration—What do we really know? Agriculture Ecosystem Environment. 2007; 118:1–5.
4. Batjes NH. Technologically achievable soil organic carbon sequestration in world croplands and grasslands. Land Degradation and Development. 2019; 30: 25–32.
5. Islam KR, Roth G, Rahman MA, Didenko NO, Reeder RC. Cover crop complements flue gas desulfurized gypsum to improve no-till soil quality. Communications in Soil Science and Plant Analysis. 2021; 52: 926-947.
6. Szostek M, Szpunar-Krok E, Pawlak R, Stanek-Tarkowska J, Ilek A. Effect of different tillage systems on soil organic carbon and enzymatic activity. Agronomy. 2022; 12: 208.
7. Islam KR, Reeder R. No-till and conservation agriculture in the United States: An example from the David Brandt farm, Carroll, Ohio. International Journal of Soil and Water Conservation. 2014; 2: 97-107.
8 Islam KR, Weil RR. Soil quality indicator properties in the mid-Atlantic region as influenced by conservation management, Journal of Soil and Water Conservation. 2000; 55: 69–78.
9. Kristensen HL, McCarty GW, Meisinger JJ. Effects of soil structure disturbance on mineralization of organic soil nitrogen. Soil Science Society of America Journal. 2000; 64: 371–378.
10. Balota EL, Filho AC, Andrade DS, Dick RP. Long-term tillage and crop rotation effects on microbial biomass and C and N mineralization in a Brazilian Oxisol. Soil and Tillage Research. 2004; 77: 137–145.
11. Aziz I, Tariq M, Islam KR. Effect of long-term no-till and conventional tillage practices on soil quality. Soil and Tillage Research. 2013; 131: 28-35.
12. Huggins DA, Reganold JP. No-till: the quiet revolution. Scientific America. 2008; 299: 70-77.

13. Kassam A, Friedrich T, Derpsch R. Global spread of conservation agriculture, International Journal of Environmental Studies. 2019; 76: 29-51.
14. Don A, Seidel F, Leifeld J, Kätterer T, Martin M, Pellerin S, Emde D, SeitzD, Chenu C. Carbon sequestration in soils and climate change mitigation—Definitions and pitfalls. Global Change Biology. 2024; 30: e16983.
15. Anonymous. No-till farming market in the U.S.: A 10-year study into the practice & trends. A custom research report prepared by the editors of Ag Equipment Intelligence with data licensed from 10 years of no-till farmer operational benchmark studies. 2023; https://www.lessitermedia.com/wp-content/uploads/AEI_No-Till-Farming-Market-in-the-US-Report_TOC_0223.pdf.
16. Weil RR, Islam KR, Stine MA, Gruver JB, Samson-Liebig SE. Estimating active carbon for soil quality assessment: A simplified method for laboratory and field use. American Journal of Alternative Agriculture. 2003; 18: 3–17.
17. Amoakwah E, Lucas ST, Didenko NA, Rahman MA, Islam KR. Impact of deforestation and temporal land-use change on soil organic carbon storage, quality, and lability. PLOS ONE. 2022; 17: e0263205.
18. Sundermeier AP, Islam KR, Raut Y, Reeder R, Dick W. Continuous no-till impacts on biophysical C sequestration. Soil Science Society of America Journal. 2011; 75: 1779-1788.
19. Ogle SM, Alsaker C, Baldock J, Bernoux M, Breidt FJ, McConkey B, Regina K, Vazquez-Amabile GG. Climate and soil characteristics determine where no-till management can store carbon in soils and mitigate greenhouse gas emissions. Science Reports. 2019; 9: 1–8.
20. Corbeels M, Marchão RL, Neto MS, Ferreira EG, Madari BE, Scopel E, Brito OR. Evidence of limited carbon sequestration in soils under no-tillage systems in the Cerrado of Brazil. Science Reports. 2016: 6: 1–8.
21. West TO, Post WM. Soil organic carbon sequestration rates by tillage and crop rotation: A global data analysis. Soil Science Society of America Journal. 2002; 66: 1930–1946.
22. Liu C, Tian J, Cheng K, Xu X, Wang Y, et al. Topsoil microbial biomass carbon pool and the microbial quotient under distinct land-use types across China: A data synthesis. Soil Science and Environ. 2024; 2: 5 doi: 10.48130/SSE-2023-0005.
23. Fan J, McConkey B, Wang H, Janzen H. Root distribution by depth for temperate agricultural crops. Field Crops Research. 2016; 189: 68–74.
24. Zhou W, Guan K, Peng B, Margenot A, Lee D, Tang J, Jin Z, Grant R, DeLucia E, Qin Z, Wander M, Wang S. How does uncertainty of soil organic carbon stock affect the calculation of carbon budgets and soil carbon credits for croplands in the US Midwest? Geoderma 2023; 429: 116254.
25. IPCC. Summary for Policymakers. *In*: Climate Change and Land: An IPCC special report on climate change, desertification, land degradation, sustainable land management, food security, and greenhouse gas fluxes in terrestrial ecosystems (P.R. Shukla, J. Skea, E. Calvo Buendia, V. Masson-Delmotte, H.-O. Pörtner, D.C. Roberts, P. Zhai, R. Slade, S. Connors, R. van Diemen, M. Ferrat, E. Haughey, S. Luz, S. Neogi, M. Pathak, J. Petzold, J. Portugal Pereira, P. Vyas, E. Huntley, K. Kissick, M. Belkacemi, J. Malley, (eds.). 2019; https:// doi. org/10.1017/ 9781009157988.001.
26. Wilson TM, Warren JG. Bulk density and carbon concentration variance influence on soil carbon stock measurements. Communications in Soil Science and Plant Analysis. 2015; 46, 2342-2356.

27. Razzaghi S, Islam KR, Ahmed IAM. Deforestation impacts soil organic carbon and nitrogen pools and carbon lability under Mediterranean climates. Journal of Soils and Sediments. 2022; https://doi.org/10.1007/s11368-022-03244-6.
28. Yelikbayev BK, Pagano MC, Mamedov MI, Islam KR. Alfalfa–organic amendments impact soil carbon sequestration and its lability in reclaimed loess. Land Degradation and Development. 2023; 35: 88-101.
29. USDA-NRCS. Soil Survey of Fairfield County, Ohio, USA. 2025; https://archive.org/details/FairfieldOH2005.
30. Islam KR, Weil RR. Microwave irradiation of soil for routine measurement of microbial biomass carbon. Biology and Fertility of Soils. 1988: 27: 408–416.
31. Anderson TH, Domsch KH. Application of eco-physiological quotients (qCO_2 and qD) on microbial biomasses from soils of different cropping histories. Soil Biology and Biochemistry. 1990; 22: 251–255.
32. Cambardella CA, Elliott ET. Particulate soil organic matter changes across a grassland cultivation sequence. Soil Science Society of America Journal. 1992; 56: 777–783.
33. Hendrix PF, Parmelee RW, Crossley DA, Coleman DC, Odum EP, Groffman FM. Detritus food webs in conventional and no-tillage agroecosystems. BioScience. 1986; 36: 374–380.
34. Feng Y, Motta AC, Reeves DW, Burmester CH, Van Santen E, Osborne JA. Soil microbial communities under conventional-till and no-till continuous cotton systems. Soil Biology and Biochemistry. 2003; 35: 1693–1703.
35. Moitinho MR, Fernandes C, Truber PV, Marcelo AV, Corá JE, da Silva Bicalho E. Arbuscular mycorrhizal fungi and soil aggregation in a no-tillage system with crop rotation. Journal of Plant Nutrition and Soil Science. 2020; doi:10.1002/jpln.201900456.
36. Kallenbach CM, Frey SD, Grandy AS. Direct evidence for microbial-derived soil organic matter formation and its ecophysiological controls. Nature Communications. 2016; 7: 13630.
37. Buchkowski RW, Shaw AN, Sihi D, Smith GR, Keiser AD. Constraining carbon and nutrient flows in soil with ecological stoichiometry. Hypothesis and theory article. Frontiers in Ecology and Evolutions. 2019; 10 Oct. doi.org/10.3389/fevo.2019.00382.
38. Gale WJ, Cambardella CA, Bailey TB. Surface residue- and root-derived carbon in stable and unstable aggregates. Soil Science Society of America Journal. 2000; 64; 196–201.
39. Gehrke C, Johanson U, Callaghan TV, Chadwick D, Robinson CH. The impact of enhanced ultraviolet-B radiation on litter quality and decomposition processes in Vaccinium leaves from the Subarctic. Oikos 1995; 72: 213.
40. Dungait JAJ, Hopkins DW, Gregory AS, Whitmore AP. Soil organic matter turnover is governed by accessibility not recalcitrance. Global Change Biology. 2012; 18: 1781-1796.
41. Mazzilli SR, Kemanian AR, Ernst OR, Jackson RB, Piñeiro G. Greater humification of belowground than aboveground biomass carbon into particulate soil organic matter in no-till corn and soybean crops. Soil Biology and Biochemistry. 2015; 85: 22-30.
42. Hubert F, Hallaire V, Sardini P, Caner L, Heddadj D. Pore morphology changes under tillage and no-tillage practices. Geoderma. 2007; 142: 226–236.
43. Barzegar AR, Yousefi A, Daryashenas A. The effect of addition of different amounts and types of organic materials on soil physical properties and yield of wheat. Plant and Soil. 2002; 247: 295-301.

44. Hassink J. The capacity of soils to preserve organic C and N by their association with clay and silt particles. Plant and Soil. 1997; 191: 77–87.
45. Powlson DS, Whitmore AP, Goulding KWT. Soil carbon sequestration to mitigate climate change: a critical re-examination to identify the true and the false. Special Issue: Soil organic matters. European Journal of Soil Science. 2011; 62: 42-55.
46. Smith P, Andren O, Karlsson T, Perala P, Regina K, Rounsevell M, van Wesemael B. Carbon sequestration potential in European croplands has been overestimated. Global Change Biology. 2005; 11: 2153-2163.
47. Luo Z, Wang E, Sun OJ. Can no-tillage stimulate carbon sequestration in agricultural soils? A meta-analysis of paired experiments. Agriculture, Ecosystem, and Environment. 2010; 139: 224-231.
48. Johnston AE, Poulton PR, Coleman K. Soil organic matter: its importance in sustainable agriculture and carbon dioxide fluxes. Advances in Agronomy. 2009; 101: 1–57.
49. Liang BC, VandenBygaart AJ, MacDonald JD, Cerkowniak D, McConkey BG, Desjardins RL, Angers DA. Revisiting no-till's impact on soil organic carbon storage in Canada. Soil and Tillage Research. 2020; 198: 104529.
50. von Haden AC, Yang WH, DeLucia EH. Soils' dirty little secret: Depth-based comparisons can be inadequate for quantifying changes in soil organic carbon and other mineral soil properties. Global Change Biology 2020; 26: 3759–3770.
51. Rovira P, Sauras-Year T, Romanyà J. Equivalent-mass versus fixed-depth as criteria for quantifying soil carbon sequestration: How relevant is the difference? Catena. 2022; 214: 106283.
