# Supplementary material for: Long-term continuous no-till corn-soybean systems: Examining soil carbon sequestration and nitrogen accumulation across various pools
Source: PLoS One. 2025 May 9;20(5):e0322891. doi: 10.1371/journal.pone.0322891 (PMC12064199; doi:10.1371/journal.pone.0322891)
Supplement: Attachment — Submitted filename: PLOS One comments to authors.docx [file pone.0322891.s003.docx]

Comments to the authors of “Long-term, Continuous No-Till Corn-Soybean Systems: Examining Soil Carbon Sequestration and Nitrogen Accumulation Across Various Pools”. While the topic is interesting, the authors need to take a major revision of the manuscript to significantly increase the readability of the study. Also, the concept of high SOC and total N with medium to long term no-tillage has been comprehensively investigated, the authors should focus on adding the novelty to this study, specifically to the introduction section. Pls consider adding a section on soil N pools as well to the introduction. The current introduction only focuses on soil C. The data analysis section should be revised, and soil depth needs to be accounted for as a repeated variable. Essentially, the statistical model should be a repeated measures repeated by soil depth. Also, the soil depth effects are missing from the tables. The authors have only presented tillage effects and interaction of tillage with depth. But the soil depth differences need to be presented (not the pooled data). The results and discussion section need to be significantly revised and reorganized. Its helpful to merge the discussion of the results along with the presentation of results. Right now, the results are all written at once and then discussed later, which is not helpful. Pls see the attached pdf file for the specific comments.
