# Supplementary material for: Long-term continuous no-till corn-soybean systems: Examining soil carbon sequestration and nitrogen accumulation across various pools
Source: PLoS One. 2025 May 9;20(5):e0322891. doi: 10.1371/journal.pone.0322891 (PMC12064199; doi:10.1371/journal.pone.0322891)
Supplement: Attachment — Submitted filename: Response to Reviewers.docx [file pone.0322891.s005.docx]

**Response to Reviewers**

The authors express their sincere gratitude to all the reviewers for their constructive and insightful comments, which have significantly improved the quality of this manuscript. Below, we provide a detailed account of the major revisions made in response to the reviewers' feedback, particularly addressing the proactive points raised by Reviewer 1.

**Response to Reviewer 1**

We thank Reviewer 1 for his/her detailed and thought-provoking comments. In response, we have made substantial revisions to the manuscript:

1. **Introduction:**
   We have rewritten the Introduction to include additional information on nitrogen (N) fractions alongside soil organic carbon (SOC), enhancing the novelty and relevance of the study. This revision emphasizes the significance of the research, conducted on one of Ohio's pioneering no-till (NT) farms. The updated introduction now provides a clearer context for how NT systems influence soil properties over time, particularly regarding microbial processes, carbon sequestration, and nitrogen accumulation.
2. **New Figure Addition:**

To reinforce the findings, we have added a new figure (Figure 2) illustrating the effects of NT on microbial biomass, basal respiration, and microbial metabolism. This figure highlights the biological mechanisms underpinning SOC sequestration under long-term NT practices.

3. **Study Novelty and Approach:**

This research uniquely evaluates medium- to long-term NT practices in temperate rainfed corn-soybean systems on farmers' fields. It investigates depth-specific impacts on SOC, total N, and labile fractions compared to conventional tillage (CT). By incorporating a gradient of NT durations (0 to 35 years) with cereal rye as a cover crop, the study explores the stoichiometric relationships between SOC sequestration and total N accumulation. It employs both fixed-depth and equivalent soil mass approaches, addressing methodological challenges often encountered in SOC and N stock assessments.

4. **Statistical Approach:**

While soil sampling was a single-event activity on farmers' fields managed under NT for varying durations, we logically treated NT and soil depth as fixed predictor variables for two-way ANOVA. Depth stratification under long-term NT is well-documented; hence, depth effects were initially excluded from the table. However, as suggested, we have now incorporated depth effects and NT × depth interactions into the analysis, presenting them in the revised tables.

5. **Results and Discussion Integration:**

As per the reviewer's recommendation, we have integrated the results and discussion sections more effectively, following a widely accepted standard approach. However, given the involvement of multiple authors from different countries in drafting the manuscript, rewriting the results and discussion in a fully integrated format would have posed significant challenges. Therefore, the results are presented first under each subheading, followed immediately by their interpretation. This widely adopted standard format ensures clarity and facilitates a comprehensive understanding of the study's outcomes for each subheading topic.

**Response to Reviewer 2**

We thank Reviewer 2 for his/her meticulous review. The following revisions were made in response to their comments:

1. **Language and Grammar:**

We conducted a thorough spell-check and corrected all grammatical and typographical errors in the manuscript.

2. **Figures:**
   All graphs have been resized and presented with high-resolution quality for improved readability and visual impact.
